# Edaphic controls of soil organic carbon in tropical agricultural landscapes

**DOI:** 10.1038/s41598-022-24655-y

**Published:** 2022-12-14

**Authors:** Jon M. Wells, Susan E. Crow, Carlos A. Sierra, Jonathan L. Deenik, Kimberly M. Carlson, Manyowa N. Meki, Jim Kiniry

**Affiliations:** 1 Department of Natural Resources and Environmental Management, University of Hawaii at Mānoa, 1910 East West Rd., Honolulu, HI 96822 USA; 2 Center for Ecosystem Science and Society, Northern Arizona University, S San Francisco St, Flagstaff, AZ 86011 USA; 3 Department of Biogeochemical Processes, Max Planck Institute for Biogeochemistry, Hans-Knöell-Str. 10, 07745 Jena, Germany; 4 Department of Ecology, Swedish University of Agricultural Sciences, Ulls Väg 16, 750 07 Uppsala, Sweden; 5 Department of Tropical Plant and Soil Sciences, University of Hawaii at Mānoa, 3190 Maile Way, Honolulu, HI 96822 USA; 6 Department of Environmental Studies, New York University, 285 Mercer Street, New York, NY 10003 USA; 7 Texas A&M AgriLife Research, Blackland Research and Extension Center, 720 East Blackland Rd, Temple, TX 76502 USA; 8 Grassland Soil and Water Research Laboratory, USDA Agricultural Research Service, 808 East Blackland Rd, Temple, TX 76502 USA

**Keywords:** Biogeochemistry, Agroecology, Climate change, Biofuels

## Abstract

Predicting soil organic carbon (SOC) is problematic in tropical soils because mechanisms of SOC (de)stabilization are not resolved. We aimed to identify such storage mechanisms in a tropical soil landscape constrained by 100 years of similar soil inputs and agricultural disturbance under the production of sugarcane, a C_4_ grass and bioenergy feedstock. We measured soil physicochemical parameters, SOC concentration, and SOC dynamics by soil horizon to one meter to identify soil parameters that can predict SOC outcomes. Applying correlative analyses, linear mixed model (LMM) regression, model selection by AICc, and hierarchical clustering we found that slow moving SOC was related to many soil parameters, while the fastest moving SOC was only related to soil surface charge. Our models explained 78–79%, 51–57%, 7–8% of variance in SOC concentration, slow pool decay, and fast pool decay, respectively. Top SOC predictors were roots, the ratio of organo-complexed iron (Fe) to aluminum (Al), water stable aggregates (WS_agg_), and cation exchange capacity (CEC). Using hierarchical clustering we also assessed SOC predictors across gradients of depth and rainfall with strong reductions in Roots, SOC, and slow pool decay associated with increasing depth, while increased rainfall was associated with increased Clay and WS_agg_ and reduced CEC in surface soils. Increased negative surface charge, water stable aggregation, organo-Fe complexation, and root inputs were key SOC protection mechanisms despite high soil disturbance. Further development of these relationships is expected to improve understanding of SOC storage mechanisms and outcomes in similar tropical agricultural soils globally.

## Introduction

Several important soil properties are typically expected to correlate with soil organic carbon (SOC) storage: soil texture^1,2^, both micro- and macroaggregates^3^, and mineralogy, like clay type, non-crystalline mineral concentration, Ca^2+^, and Mg^2+^^4–7^. These soil properties vary across the landscape as a product of soil forming factors that include time, climate, parent material, topography, and biota^8,9^. Mineralogy has been well associated with SOC stabilization in the long term, with the inherently low surface areas of 1:1 silicate clays linked to limited sorptive capacity and shorter SOC storage timescales^10^. In contrast, 2:1 clays like smectite have been shown to have longer SOC retention times^11^. Torn et al.^4^ also suggest that non-crystalline mineralogy like amorphous Fe and Al hydroxides, which accumulate in weathered soils, can retain SOC for millennia. However, there is still need to disentangle long-term mineral driven SOC storage from less persistent short-term SOC kinetics and storage that is thought to be controlled by the equilibrium of soil organic matter (SOM) inputs and outputs^12^.

Despite the many controlling relationships expected between soil physicochemical parameters and SOC storage and dynamics, many global ecosystem models^13–16^ have estimated SOC without strong data-driven relationships between soil physicochemical parameters and SOC (de)stabilization. This contributes to a gap between simulation of SOC and accurate representation of soil mechanisms and processes^17,19^. Global predictive SOC models such as CENTURY^15^, RothC^13^, Biome-BGC^14^, and CASA^16^ are based on climate or land use change and are typically derived from over-simplified or purely conceptual relationships between soil parameters and SOC. However, there have been important improvements to older models, such as the inclusion of Iron (Fe) and aluminum (Al) in a RothC based model for Japanese Andosols which found improved model predictions using detailed soil mineralogy^20^. Further improving model accuracy will require development of generalizable relationships between soil physicochemical parameters and SOC protection and stabilization beyond clay modifiers^21^.

There is also a lack of synthesis of major drivers of SOC flow and storage across diverse soils^21,22^, especially in the tropics. Establishing better predictive relationships will thus require more detailed and mechanistic conceptual models^23^, as well as better aggregation and dissemination of existing tropical soil data^22^. However, Powers et al.^24^ found only 80 usable studies for their analysis of biophysical controls of SOC stocks across tropical land use, with findings indicative of high uncertainty in SOC changes based on land use change. Thus, improving both statistical predictive models and mathematical system models will require better mechanistic understanding of SOC storage and more-detailed data in the tropics.

Movement towards empirically fit SOC dynamics models, like conceptual models built in SoilR and fit to soil fraction or soil incubation data^18^, can further inform SOC kinetics and subsequently improve tropical SOC modeling capabilities. As time is not well accounted for in current models of SOC, identification of how SOC protection mechanisms control the rate of (de)stabilization of SOC through time must be resolved. Geospatial use of sound SOC sub-component models will also require connections between mechanism of SOC storage and predictive soil physicochemical parameters that can be measured across the landscape. Thus, to support and refine models of SOC storage and dynamics in tropical agricultural areas we investigated relationships between edaphic controls and SOC content/kinetics across a constrained landscape that experienced 100 years of similar agricultural input and disturbance. Greater understanding of mechanisms driving SOC response in disturbed tropical soils could allow this and other tropical agricultural areas to sustainably transition away from sugar production towards combinations of diversified agriculture, agroforestry, and biofuel feedstocks.

## Methods

### Study area and soil collection

**Figure 1:**
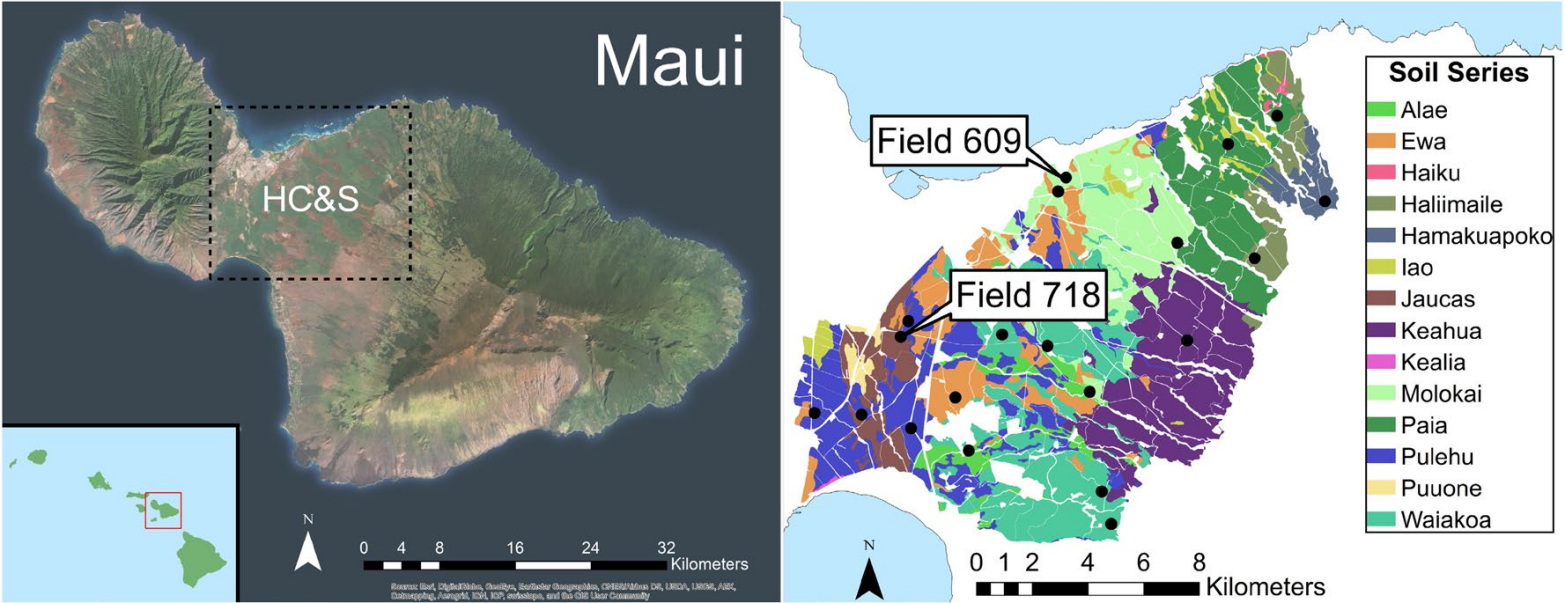
Hawaiian Commercial and Sugar in central Maui with main Hawaiian Islands inset (left). Soil series identified by NRCS across HC&S fields (right) with black dots indicating 20 locations where soils were sampled to test landscape level differences in topical soil kinetics and associated soil properties under conventional sugarcane. Maps from Ref.^19^ created using ESRI ArcGIS with soil series data from: Soil Survey Staff, Natural Resources Conservation Service, United States Department of Agriculture, Web Soil Survey, Available online at http://websoilsurvey.nrcs.usda.gov/. Accessed [07/30/2016]^19^.

Twenty NRCS map units were selected across Hawaii Commercial & Sugar Company (HC&S) in central Maui that represented seven soil orders, 10 NRCS soil series, and approximately 77% of the total plantation area (Fig. 1). Soil heterogeneity across the landscape allowed for the comparison of a continuum of soil and soil properties that have experienced the same C_4_ grass inputs and agricultural treatment under sugarcane production for over 100 years. Conventional sugarcane production involved 2-year growth followed by harvest burn, collection of remaining stalks by mechanical ripper, deep tillage to 40 cm, no crop rotations, and little to no residue return. The sampled soils, collected from September-August 2015, thus represent a baseline of SOC after input-intensive tropical agriculture and long-term soil disturbance. Elemental analyses from this work show consistent agricultural disturbances led to degraded SOC content ranging from 0.23 to 2.91% SOC of soil mass with an average of only 1.16% SOC across all locations and depths.

The homogenized land use history allowed focused investigation of soil property effects on SOC storage across heterogenous soils (Table 1). Though soil inputs (e.g. water, nutrients, root inputs, residue removal) and disturbance regimes (e.g. burn, rip, till, compaction, no crop rotation) were consistent across the 20 field locations, average annual surface temperatures varied from 22.9 to 25.1 °C with a mean of 24.4 °C, average annual relative humidity varied from 70.4 to 79.2% with a mean of 73.4%, and average annual rainfall varied from 306 to 1493 mm with a mean of 575 mm. Gradients of rainfall, relative humidity, and elevation across the site generally increase in an east/north-east direction towards the prevailing winds and up the western slope of Haleakalā. In contrast, surface temperatures increase in the opposite direction towards Kihei and the southern tip of the West Maui Mountains.

**Table 1:** NRCS soil classification and environmental conditions at 20 field sites.

NRCS series^a^	Taxonomic class name^a^	Elevation^b^ (m)	Temperature^b^ (°C)	Rainfall^b^ (mm)	RH^1^ (%)
Alae	Sandy or sandy-skeletal, mixed, isohyperthermic Ustic Haplocalcid	126	24.36	414.21	74.22
		33	24.97	305.61	71.10
Ewa	Fine, kaolinitic, isohyperthermic Aridic Haplustoll	23	24.98	468.85	70.69
		40	24.92	335.87	71.31
Haliimaile	Very-fine, parasesquic, isothermic Oxic Dystrustept	264	23.28	1112.53	78.37
Hamakuapoko	Fine, mixed, semiactive, isohyperthermic Andic Palehumult	306	22.89	1492.86	79.18
Jaucas	Carbonatic, isohyperthermic Typic Ustipsamment	46	24.85	394.84	71.62
Keahua	Fine, kaolinitic, isohyperthermic Ustic Haplocambids	205	23.81	642.57	76.61
Molokai	Very-fine, kaolinitic, isohyperthermic Typic Eutrotorrox	123	24.27	686.56	74.27
		21	24.98	474.26	70.66
Paia	Very-fine, parasesquic, isohyperthermic Torroxic Haplustoll	103	24.24	1193.53	73.46
		92	24.36	987.25	73.26
Pulehu	Fine-loamy, mixed, semiactive, isohyperthermic Cumulic Haplustoll	44	24.86	407.02	71.53
		71	24.72	432.86	72.29
		33	24.96	346.82	71.08
		16	25.08	316.54	70.44
Waiakoa	Fine, kaolinitic, isohyperthermic Torroxic Haplustoll	188	23.96	345.87	76.15
		204	23.86	333.56	76.63
		63	24.74	399.17	72.24
		85	23.22	420.71	73.24

^a^Soil descriptions^26^.

^b^Interpolated estimates from Ref.^25^.

### Soil sampling and analysis

Pit locations were identified with a handheld GPS and were sampled using NRCS Rapid Carbon Assessment methods^27^. A total of 75 horizons were identified from the 20 selected map units to a depth of 1 m^28,29^. The central depth of each horizon was sampled using volumetric bulk density cores up to 50 cm. After 50 cm, a hand auger was used to check for any further horizon changes. The bulk density of horizons past 50 cm were estimated using collected soil mass and known auger size. Collected soils were air dried, processed through a 2 mm sieve, and analyzed for total C and nitrogen percent, SOC percent, soil texture, iron (Fe) and aluminum (Al) minerals, pH, cation and anion exchange capacity, extractable cations, wet and dry size classes, aggregate stability, and soil water potential at -15 kPa. Total C and nitrogen were measured by elemental analysis (Costech, ECS 4010, Valencia, CA), with SOC content determined by elemental analysis after hydrochloric acid digestion to remove carbonates. Soil texture was measured using sedimentary separation, while a 10:1 soil slurry in water was used to test soil pH. Soil pressure plates were used to measure soil water potential at -15 kPa.

Fe and Al oxides were quantified in mineral phases using selective dissolutions of collected soils, including: (1) a 20:1 sodium citrate to sodium dithionite extraction, shaken 16 h, to quantify total free minerals^30^, (2) 0.25 M hydroxylamine hydrochloride and hydrochloric acid extraction, shaken 16 h, to quantify amorphous minerals^31^, and (3) 0.1 M sodium pyrophosphate (pH 10), shaken 16 h and centrifuged at 20,000*g*, to quantify organo-bound metals^30^. Extracted Fe, Al, and Si from al extractions were measured by inductively coupled plasma analysis (PerkinElmer, Optima ICP-OES, Norwalk, CT). Exploratory ratios of Fe/Al, Fe/Si, and Al/Si for the citrate/dithionite (c), hydroxylamine (h), and pyrophosphate (p) extractions were calculated. Crystalline Fe, operationally-defined as the difference between the citrate dithionite and hydroxylamine extraction, and Al + ½ Fe^32^ were calculated for each extraction.

Plant-available phosphorus was extracted by the Olsen method using 0.5 M sodium bicarbonate adjusted to pH 8.5 and measured by continuous flow colorimetry (Hach, Lachat Quickchem 8500, Loveland, CO). Exchangeable cations (i.e. calcium, magnesium, potassium, and sodium), effective cation exchange capacity, and anion exchange capacity were measured by compulsive exchange using barium chloride and magnesium sulfate^33^. Cations were quantified by continuous flow colorimetry and flame-spectroscopy (Hach, Lachat Quickchem 8500, Loveland, CO). Field soils were air dried and initially passed through a 2 mm sieve before size classes of macroaggregate (2 mm – 250 µm) and microaggregate (< 250 µm) were separated using a 250 µm sieve. Further wet sieving at 250 µm was conducted on 4 g of dry sieved macroaggregates using a wet sieving apparatus (Eijkelkamp, Wet Sieving Apparatus, Morrisville, NC). In short, dry sieved macroaggregates were wet sieved at 60 oscillations a minute for 45 min in distilled water. Soil particles passing through the 250 µm sieve were classified as non-water-stable aggregates, while soil particles retained were dispersed by 16 h of shaking in 2% sodium hexametaphosphate. After soil dispersion, roots and rocks were separated from remaining soil on a 250 µm sieve using distilled water and light agitation with a rubber policeman. Roots were further separated from rocks using a combination of density separation in water and visual removal with tweezers. Though the Roots metric technically includes particulate organic matter (POM), most recovered material was visually identifiable as root biomass.

### Soil incubation

The equivalent of 50 dry grams of soil were incubated for 90 days at 25 °C in 500 mL septa capped cell culture bottles after moisture adjustment to -15 kPa moisture content using soil pressure plates^28,29^. Evolved gases were sampled using 10 mL syringes, stored in evacuated 3 mL Exetainers (Labco Limited, Lampeter, UK), and analyzed by GC (Shimadzu, GC-2010 Green House Gas analyzer, Kyoto, Japan) using a 4-point calibration curve of mixed CO_2_, N_2_O, and CH_4_ standard gas. After each sampling event, incubation bottles were flushed with air, resealed, sampled for initial concentration, and returned to controlled environment chambers (Caron, 7000-33). All incubations were sampled on the same interval: day 0, 2, 4, 6, 8, 10, 12, 14, 17, 20, 23, 26, 30, 35, 40, 45, 50, 55, 60, 65, 70, 75, 80, 85, 90. The mass of CO_2_ evolved from the soil in each incubation chamber was calculated with the gas law using the measured concentration, known volume, and Standard Ambient Temperature and Pressure (SATP). The mass of C from CO_2_ (C-CO_2_) was normalized by the dry weight of soil in each chamber and cumulative flux curves of were tracked throughout the 90-day incubation experiment. C pools and fluxes were estimated using compartment models fit to cumulative C–CO_2_ incubation curves as they describe C mineralization kinetics of soils ^18^.

### Modeling soil carbon pools using SoilR

**Figure 2:**
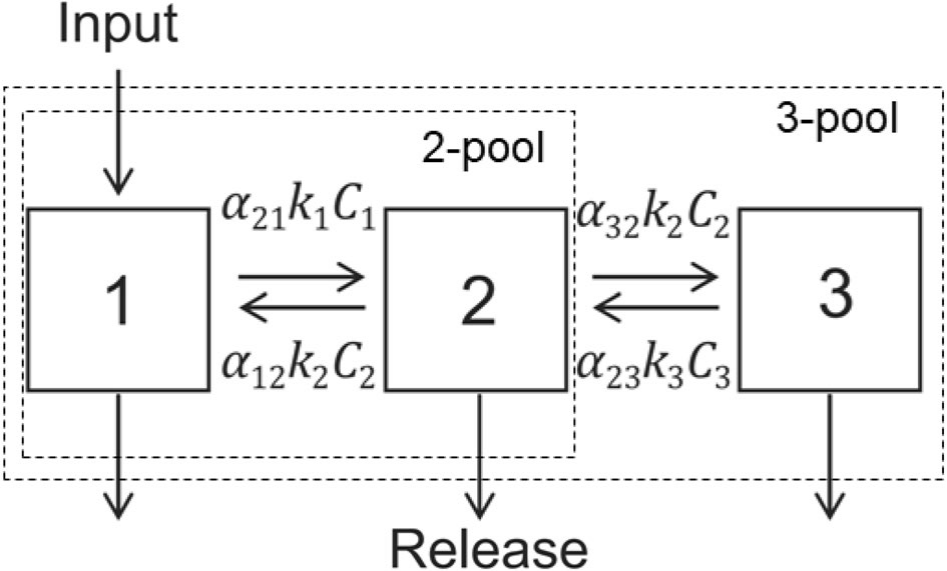
Conceptual 2- and 3-pool models with series C flow, feedback between pools, and respiration loss from all pools.

To estimate kinetic SOC fractions, Bayesian solutions of two- and three-pool compartment models (Fig. 2) were implemented in R. Models were fit to cumulative C-CO_2_ curves using the SoilR package and a generalized model of linear dynamical systems^18^:

1 $$\frac{d{\varvec{C}}\left(t\right)}{dt}={\varvec{I}}\left(t\right)+{\varvec{A}}\left(t\right)\cdot {\varvec{C}}\left(t\right),$$

where *I*(t) is a time-dependent column vector of inputs to each compartment *n*, *A*(t) is a *n* × *n* square matrix that contains compartment decomposition rates and transfer coefficients between compartments, and *C*(t) is a *n* × 1 vector of C compartment size (pools) at a given time t. Three-pool SOC models can be expressed generically by a system of differential equations of the form:

2 $$\left(\begin{array}{c}\begin{array}{c}\begin{array}{c}\begin{array}{c}{dC}_{1}/dt\\ {dC}_{2}/dt\end{array}\\ {dC}_{3}/dt\end{array}\end{array}\end{array}\right)=\left(\begin{array}{c}\begin{array}{c} {I}_{1}\\ 0\end{array}\\ 0\end{array}\right)+\left(\begin{array}{ccc}{-k}_{1}& {\alpha }_{\mathrm{1,2}}{k}_{2}& 0\\ {\alpha }_{\mathrm{2,1}}{k}_{1}& {-k}_{2}& {\alpha }_{\mathrm{2,3}}{k}_{3}\\ 0& {\alpha }_{\mathrm{3,2}}{k}_{2}& {-k}_{3}\end{array}\right)\cdot \left(\begin{array}{c}\begin{array}{c}\begin{array}{c} {C}_{1}\\ {C}_{2}\end{array}\end{array}\\ {C}_{3}\end{array}\right).$$

With initial conditions:

3 $$\left(\begin{array}{c}{C}_{1i}\\ {C}_{2i}\\ {C}_{3i}\end{array}\right)={C}_{i}\cdot \left(\begin{array}{c}{\gamma }_{1}\\ {\gamma }_{2}\\ {\gamma }_{3}\end{array}\right),$$

where *k*_*i*_ represents the decay rate in each pool, *α*_*i,j*_ represents the transfer rate of decayed C in pool *j* that flows to pool *i*, while γ_*i*_ represents the proportion of C in each pool, and *I*_*i*_ represents the input to each compartment. Two-pool models take the same form as Eqs. (2) and (3), except the third row of every matrix, and third column of the *A*(t) matrix, are removed. In this sense these matrix models can be scaled up or down to include as many compartments as desired, with the caveat that complex models with many compartments and transfers can become untenable.

Equation (1) was thus fit to the cumulative CO_2_ curve of each soil sample using a two-step parameter optimization involving initial classical optimization with a Nelder-Mead algorithm^34^ followed by Bayesian optimization using a Markov Chain Monte Carlo (MCMC) procedure^35^. Both optimizations were conducted in R^36^ using a combination of the FME^35^ and SoilR^18^ packages.

Collinearity analysis indicated that two-pool models were the least collinear (i.e., the most likely to result in meaningful solutions) but would still require fixing at least two parameters. From the collinearity analysis, the best two-pool models used pool sizes (γ_1_, γ_2_) and decay rates (*k*_1_, *k*_2_), but required that the transfer rates (α_2,1_, α_1,2_) be fixed. To address this issue, we used the transfer rates from a soil fraction-based model developed by Crow et al.^37^ at one of the 20 sites investigated in this study. The transfer rates, which describe the amount of decayed C that flows through any given transfer path, are difficult to quantify and would require soil density fractionation and fraction-based compartment models, or controlled input and labeling experiments, to quantify, which have not been conducted. Thus, models were informed with best-available data by fixing the forward transfer rate (α_2,1_) and backward transfer rate (α_1,2_) at 0.85 and 0.15, respectively^37^.

The resulting kinetic SOCC fraction estimates (i.e., pool sizes and decay rates) are framed here as dependent variables that respond to differences in independent soil physicochemical parameters measured across this agricultural landscape. The slow pool decay rate (k_2_) and fast pool decay rate (k_1_) from two-pool models are presented in comparison to SOC. To discuss only the most identifiable solutions, the 3-pool models were not presented, and only 2-pool model results were used.

### Linear mixed models and model selection

Several statistical models were created to investigate relationships between SOC content, SOC decay rates, and soil physicochemical properties (Table 2) that may promote SOC persistence despite long-term agricultural disturbance. For each C response variable (SOC, k_2_, and k_1_), three sequential linear mixed models (LMM), with pedon as a random effect, were constructed: (1) using no fixed effect (intercept-only; equivalent to a random effects ANOVA), (2) using only physicochemical properties as fixed effects that showed significant spearman’s ρ to the conditional residuals of the intercept-only LMM, and (3) a final model where root-mineral interaction terms and physicochemical properties with significant spearman’s ρ were further reduced by model selection using the Akaike Information Criterion for small sample sizes (AICc).

**Table 2:** Potential physicochemical soil predictors of SOC content, k_2_, k_1_ in linear mixed models.

Parameter	Analysis	Interpretation
Fe_c_	Citrate/dithionite-extractable Fe (mg g^-1^)	Total free oxides
Al_c_	Citrate/dithionite-extractable Al (mg g^-1^)	
Si_c_	Citrate/dithionite-extractable Si (mg g^-1^)	
Fe_h_	Hydroxylamine/HCl-extractable Fe (mg g^-1^)	Amorphous oxyhydroxides
Al_h_	Hydroxylamine/HCl-extractable Al (mg g^-1^)	
Si_h_	Hydroxylamine/HCl-extractable Si (mg g^-1^)	
Fe_p_	Sodium Pyrophosphate-extractable Fe (mg g^-1^)	Organo-complexed metals
Al_p_	Sodium Pyrophosphate-extractable Al (mg g^-1^)	
Si_p_	Sodium Pyrophosphate-extractable Si (mg g^-1^)	
Fe_c-h_	Fe_c_–Fe_h_ (mg g^-1^)	Crystalline Fe oxides
Al_c_ + 0.5 Fe_c_	Al + 0.5 Fe within each extraction (mg g^-1^)	“reactive metal”, approximately normalized by atomic mass of Al and Fe^32^
Al_h_ + 0.5 Fe_h_		
Al_p_ + 0.5 Fe_p_		
$\frac{{Fe}_{p}+{Si}_{p}}{{Fe}_{p}+{Si}_{p}+{Al}_{p}}$	Organo-Fe and organo-Si normalized by total organo-complexes	Fraction of more stable Fe and Si organo-complexes
Fe_c_/Al_c_	Citrate/dithionite-extractable Fe/Al ratio	Total free oxide Fe/Al ratio
Fe_h_/Al_h_	Hydroxylamine/HCl-extractable Fe/Al ratio	Amorphous Fe/Al ratio
Fe_p_/Al_p_	Sodium pyrophosphate-extractable Fe/Al ratio	Organo-complexed Fe/Al ratio
Si_c_/Al_c_	Citrate/dithionite-extractable Si/Al ratio	Total free oxide Si/Al ratio
Si_h_/Al_h_	Hydroxylamine/HCl-extractable Si/Al ratio	Amorphous Si/Al ratio
Si_p_/Al_p_	Sodium pyrophosphate-extractable Si/Al ratio	Organo-complexed Si/Al ratio
Fe_c_/Si_c_	Citrate/dithionite-extractable Fe/Si ratio	Total free oxide Fe/Si ratio
Fe_h_/Si_h_	Hydroxylamine/HCl-extractable Fe/Si ratio	Amorphous Fe/Si ratio
Fe_p_/Si_p_	Sodium pyrophosphate-extractable Fe/Si ratio	Organo-complexed Fe/Si ratio
Depth	Continuous measurement (cm)	
BD	Bulk density (g cm^-^^3^)	
pH	Soil pH	
Phos	Phosphorus (mg kg^-1^)	
Clay	Particles < 2 µm (%)	
Silt	Particles 2–50 µm (%)	
Sand	Particles 50–2000 µm (%)	
Clay + Silt	Particles < 50 µm (%)	
Macro_agg_	Dry sieved macro-aggregates 250–2000 µm (%)	
Micro_agg_	Dry sieved micro-aggregates < 250 µm (%)	
WS_agg_	Water stable macro-aggregates 250–2000 µm (%)	
NWS_agg_	Non-water stable macro-aggregates 250–2000 µm (%)	
Roots	Roots (%)	Roots + particulate organic matter
Rocks	Rocks (%)	
WC_15kPa_	Estimate of field capacity of soils at -15 kPa (%)	Water held by dry soil at field capacity
CEC	Cation exchange capacity at field pH (cmol_c_ kg^-1^)	Soil surface charge
AEC	Anion exchange capacity at field pH (cmol_c_ kg^-1^)	
Ca	Extractable calcium (mg g^-1^)	
K	Extractable Potassium (mg g^-1^)	
Na	Extractable sodium (mg g^-1^)	
Mg	Extractable magnesium (mg g^-1^)	

Due to computational and theory-based restrictions on model selection, it was necessary to first reduce the possible independent variables (i.e., soil physicochemical properties) to only those that have prediction potential before performing model selection. This was accomplished using the under-defined models, i.e. LMM(1), of each C response variable (SOC, *k*_2_, and *k*_1_) and identifying which physicochemical properties showed spearman correlation to conditional model residuals, like Rasmussen et al.^21^. Soil properties that could explain residual model variance were thus candidates for final model predictors. The conditional residuals were also parsed above and below the median of each climate variable (i.e., rainfall, humidity, and surface temperature) to evaluate climate effects. Correlation significance was assessed using Bonferroni’s corrected α (0.05/308 = 0.000162), where 308 is the number of correlations conducted for each response variable. The correlative analyses allowed visualization of potential relationships between C response variables and physicochemical properties, as well as how climate may mediate these relationships.

Using this information allowed LMM (2) to be built with physicochemical properties that explained some residual model variance. For each response variable, LMM (2) was developed for the sole purpose of creating a global model for model selection by AICc. Interpreting LMM (2) of each response variable is not very productive as many soil metrics are collinear and a model with this many parameters will suffer from model overfitting. Model selection by AICc was thus chosen to identify combinations of predictors that could best-explain C response while avoiding overly complex models and overfitting.

For parsimony, and under the assumption that similar soil properties should control both the decay rates and overall SOC outcomes, a unified list of significant factors across the intercept-only models of SOC, k_2_, and k_1_ was created as the basis of the third and final model, LMM (3). Root-mineral interaction terms were added to LMM (3) before model selection based on a-priori expectations that roots and mineralogy are major mediators of C input and storage in soils^38^. Models were selected using AICc in R using the dredge function in the MuMIn package^39^. Due to the high correlation between many soil properties (e.g., roots and depth, depth and mineralogy, mineralogy and mineral ratios, etc.), the model selection process for SOC also selected between highly correlated predictors to avoid confounding regression coefficient estimates. This was accomplished using logical subsetting in the dredge function of the MuMIn package to eliminate nested models where both correlated predictors occurred.

Climate variables are not included in any LMM. Instead, climate variables are framed as soil forming factors that established soil physicochemical properties and not as direct drivers of SOC storage or decay. For the same reason that collinear soil properties should not be included in a single model, including climate variables that correlate with soil physicochemical properties (e.g., rainfall and clay) will confound regression coefficient estimates. Further, we assumed that direct climate influence on SOC have been constrained by consistent agricultural practices, mono-cropping, and landscape-scale irrigation that have normalized C inputs and losses over 100 years of sugarcane cultivation. We also expect that agricultural disturbance has reduced SOC to minimal levels with remaining C largely persisting due to physicochemical protection mechanisms like aggregation, organo-mineral complexation, and organo-metal ligand bonding. The moisture-adjusted and temperature-controlled incubation also removed direct influence of climate on comparative measurement of SOC dynamics across soil horizons. With these assumptions, we focused on soil physicochemical properties as key controls of SOC and dynamic C response in this system.

### Hierarchical clustering to evaluate depth and rainfall gradients

Visualization of relationships within the data between C responses and best predictors by depth and rainfall was accomplished using agglomerative hierarchical clustering. The clustering algorithm used Euclidean distance measures and Ward’s minimum variance method as implemented in the hclust function within the Stats package in R^36^. Unscaled C responses, final model predictors, and depth data were clustered by minimizing total within-cluster variance, where additional clusters beyond the fifth showed little reduction in variance. The clustering procedure was repeated for surface soils by subsetting C response and final model predictors to only the topsoil, adding associated rainfall data, and clustering again based on minimized within-cluster variance. In the case of surface soils clustered by rainfall, additional clusters past the third showed little reduction in variance. Both clustering procedures (i.e., depth and rainfall) used unscaled data to take advantage of the numerically higher values of depth (0–100 cm) and rainfall (306–1493 mm) compared to other data types. This negated the need to weight or scale the data and resulted in parameters clustered predominantly by the depth or rainfall gradient, respectively. Clear separation of clusters by depth and rainfall were found, which allowed for interpretation of the relationships between C response, soil physicochemical predictors, and depth and rainfall gradients.

## Results and discussion

### Physicochemical correlates to SOC, k_2_, and k_1_

Conditional residuals of intercept-only LMMs describing SOC content, k1, and k2 response were used to assess which soil physicochemical properties can explain SOC response across the measured landscape and how climate-induced changes in soil physicochemical development may mediate these relationships. Overall, pool and flux data showed fewer significant correlations to soil physicochemical properties and weaker correlations in general compared to those found between total SOC content and physicochemical soil properties across the landscape and through depth. Reduced correlation between dynamic C response and soil physicochemical properties may show a disconnect between the strength of physicochemical controls on total SOC content compared to dynamic SOC pools.

**Figure 3:**
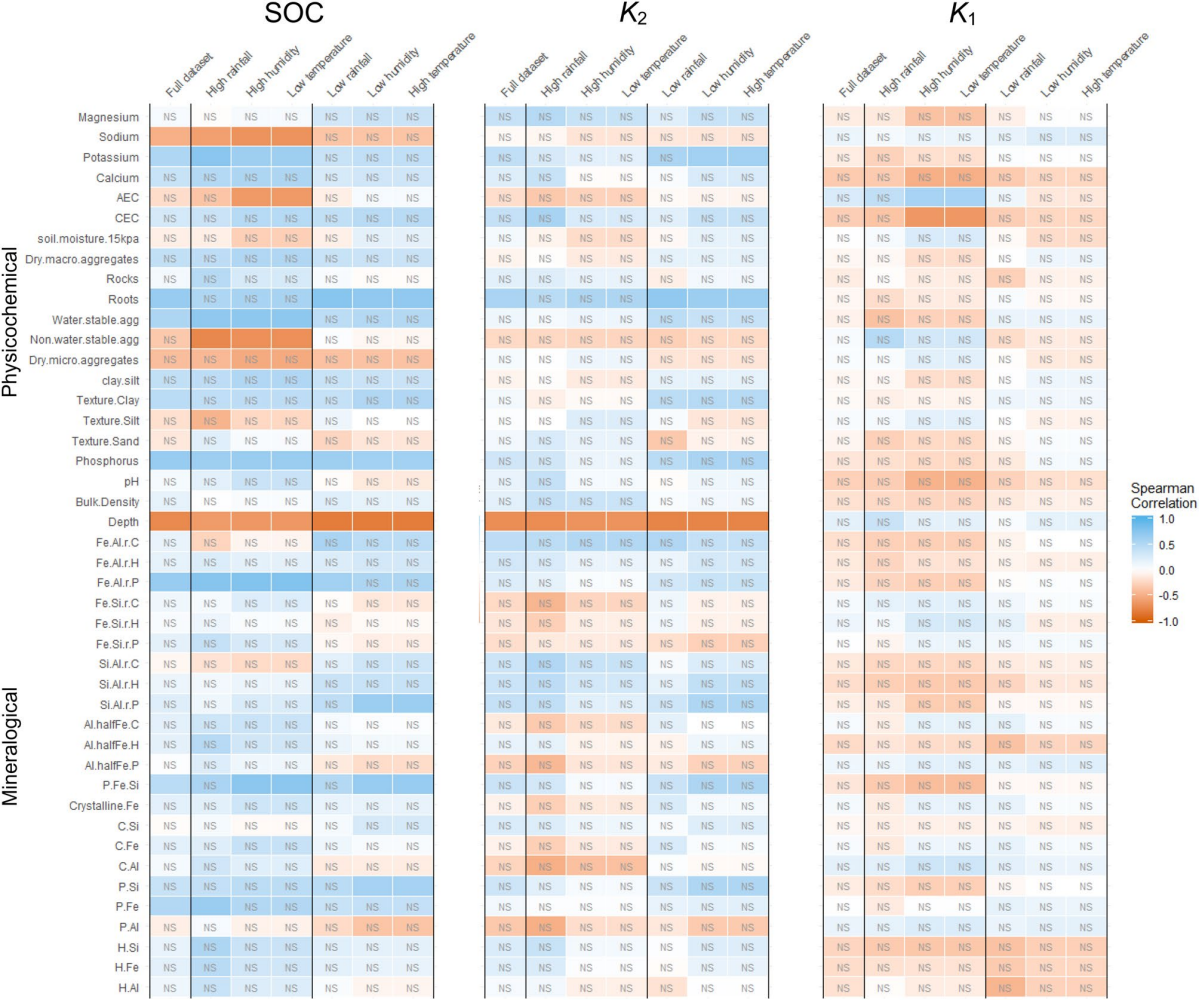
Physicochemical soil properties correlated to conditional residual variance within intercept-only linear mixed models of SOC content, the rates of fast pool C decay (k1) and slow pool C decay (k2). Residuals from LMM (1) for SOC, k_1_, and k_2_ were parsed by climate, above and below the median of each climate variable, and assessed for spearman correlation to measured soil physicochemical properties. Correlations within a response variable that are greater than the Bonferroni adjusted α of all comparisons (0.05/308 = 0.000162) are labeled as non-significant (NS).

In areas of high rainfall, high humidity, and low temperature SOC was positively correlated with phosphorus, water stable aggregates, potassium, Fe_p_, Fe_p_/Al_p,_ and (Fe_p_ + Si_p_)/(Fe_p_ + Si_p_ + Al_p_) (Fig. 3). Sodium, AEC, and non-water stable aggregates were negatively correlated with SOC. In contrast, SOC was positively correlated with roots, phosphorus, and Si_p_, and the Si_p_/Al_p_ ratio in low rainfall, low humidity, and high temperature areas. Depth was also negatively correlated with SOC in both wetter/cooler and dryer/hotter systems but showed highest correlation in dryer areas. However, the correlation between SOC and depth was reduced in areas of high rainfall. This could be an indication that depth is not a consistent predictor of SOC across the climate gradients present in this system, which could be a consequence of greater soil weathering and deeper profile development in wetter areas.

In contrast to SOC content, both slow pool (k_2_) and fast pool (k_1_) decay rates have few potential physicochemical predictors with expected explanatory power. In the fast pool, the only signal above the Bonferroni cut-off was soil surface charge, as measured by AEC and CEC. Residuals of the k_1_ intercept-only LMM suggest that AEC increases are associated with increased fast pool loss, while CEC increases have the opposite effect. Slow pool decay instead shows positive correlation with roots, potassium, and the accumulation of total Fe compared to Al (Fe_c_/Al_c_), with negative correlation with depth. This correlation procedure thus does not show a clear mechanism for k2 differences. Instead, the correlations seem to suggest that more root C means more overall loss, which may suggest proportional decay based on simple kinetics like pool size and temperature. These potential relationships were more formally tested using a LMM framework and model selection.

### Generalized physicochemical predictors of SOC, k_2_, and k_1_

**Figure 4:**
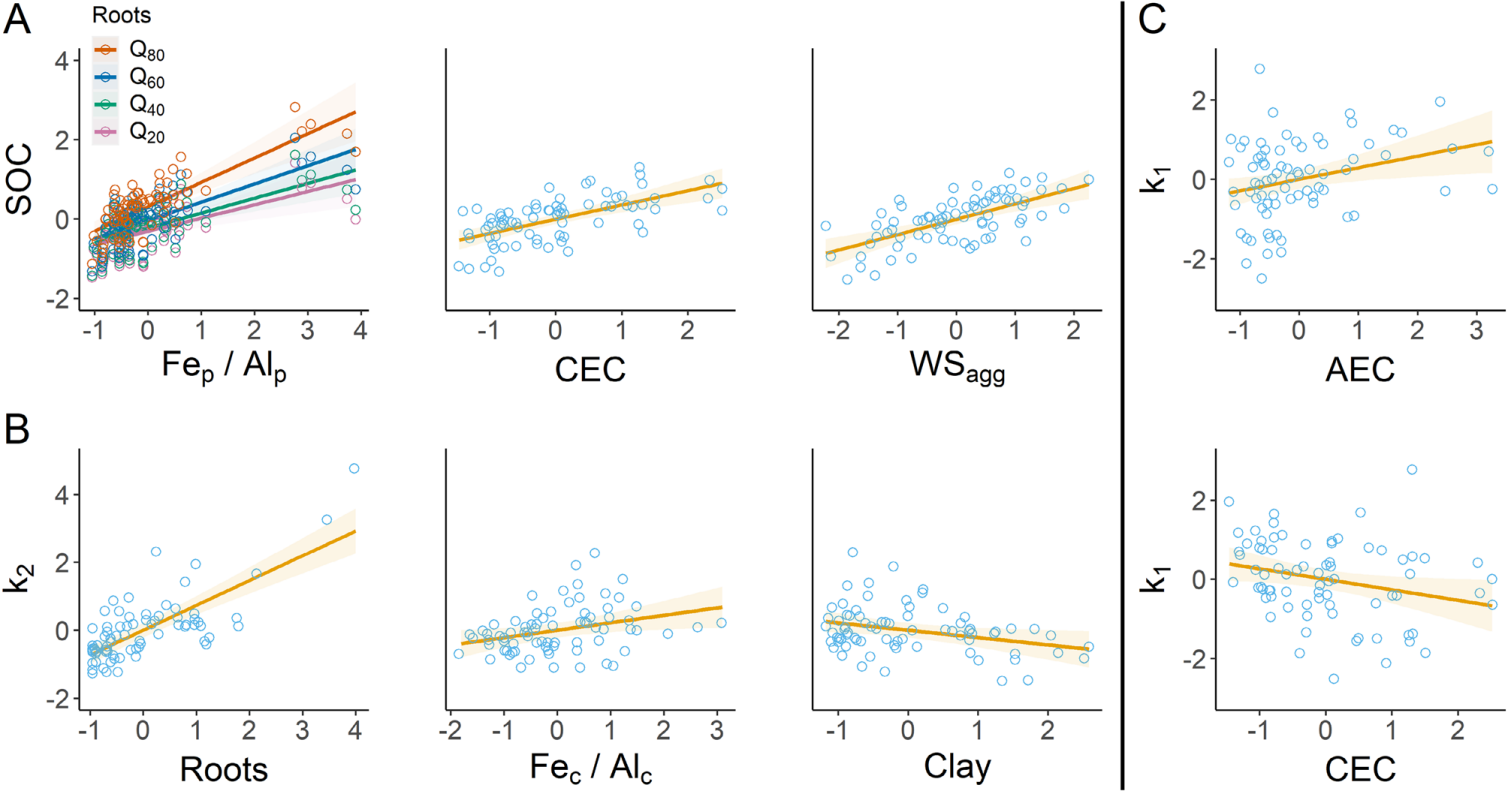
Partial regression plots of C response predicted by physicochemical soil properties across 20 field locations sampled by horizon to 1 m. Models with the highest marginal R^2^ after model selection by AICc are visualized for each C response variable: SOC content (**A**), the slow pool decay rate, k2 (**B**), and the fast pool decay rate, k1 (**C**). All data were mean-centered and scaled by relative standard deviation for comparison. Regression coefficients are shown as fitted lines with 95% confidence intervals.

Model selection of SOC content, k_2_, and k_1_ using AICc resulted in several top models within two AICc points (Table 3), indicating they were not separable by information theory. AICc based model selection showed that CEC, WS_agg_, Roots, and Fe_p_/Al_p_ were strong predictors of SOC across soil orders, depths, and climate, meaning these predictors were generalizable across these gradients in this system (Fig. 4). In comparison, slow pool decay rates were less predictable by physicochemical properties, with roots, Fe_c_/Al_c_, and percent clay most predictive based on AICc and marginal R^2^. In contrast to SOC and slow pool decay rates, the fast pool decay rate was least related to physicochemical properties and only marginally predicted by soil surface charge, where CEC and AEC showed small opposing effects on fast pool decay rates across all horizons. Physicochemical properties in top SOC models explained 78–79% of the variance through depth and across the landscape, while models of k_2_ and k_1_ explained only 51–56% and 7–8% of variance, respectively. Overall, measured physicochemical properties described SOC content better than SOC decay, with slow pool decay rates better predicted than the fast pool decay rates describing the most labile C fractions.

**Table 3:** Model selection results for SOC content, k_2_, and k_1_ with associated marginal (fixed effect) R^2^, conditional (fixed and random effect) R^2^, and AICc. Models within 2 AICc points for each C response are presented.

Response	Model terms	Marginal R^2^	Conditional R^2^	AICc
SOC (1) (3.1)	CEC, WS_agg_, Fe_p_/Al_p_, Roots	0.780	0.816	130.2
SOC (2) (3.2)	CEC, WS_agg_, Fe_p_/Al_p_, Roots, Fe_p_/Al_p_: Roots	0.791	0.812	131.3
k_2_ (1)	Roots, Si_p_/Al_p_	0.544	0.687	166.6
k_2_ (2)	Roots, Fe_c_/Al_c_	0.531	0.729	166.9
k_2_ (3)	Roots	0.510	0.696	167.6
k_2_ (4)	Roots, Clay	0.552	0.655	167.7
k_2_ (5)	Roots, Fe_c_/Al_c_, Clay,	0.568	0.706	167.8
k_1_ (1)	AEC	0.083	0.137	218.9
k_1_ (2)	CEC	0.068	0.115	220.4

The difference in variance explained by physicochemical soil properties across C response suggests several possibilities: (1) we have not measured or identified all important physicochemical controls of SOC dynamics, (2) physicochemical metrics are not strongly predictive of dynamic SOC response, which may be better predicted by biological and enzymatic investigations^40^, and/or (3) the difference in time-scale from a bulk SOC measurement developing over years to fast pool losses over a few weeks may lead to greater stochasticity and fewer predictable relationships. Though the data does not speak to why relationships between soil physicochemical parameters are worse for fast cycling C, it does suggest that slower C response is more related to pedogenic soil properties, and inversely that changes in fast C decay are largely decoupled from the soil properties that predict differences in long-term SOC outcomes. However, it is not resolved how a small effect could compound to increase SOC over long time periods and large amounts of soil.

Soil physicochemical parameters that were most predictive of SOC across gradients of depth and climate indicate several potential SOC stabilization mechanisms. SOC increased in accordance with WS_agg_ and suggests that physical protection is an important mechanism promoting SOC storage in this consistently wetted and disturbed system. Potential mechanisms behind CEC and SOC relationships are more complicated. SOC increased with CEC, which suggests either increased adsorption of positively charged SOC on negatively charge soil surfaces, increased cation bridging, direct increases in CEC based on negatively charged SOC side chains, or some combination of these processes. Models of k_1_ indicated that increased CEC was associated with reduced fast pool loss and inversely that decreased AEC was associated with reduced fast pool loss. Agreement between SOC and k_1_ outcomes based on CEC suggests that increased negative surface charge can shunt SOC away from microbes and onto soil surfaces to reduce C turnover. In contrast, roots as a top SOC predictor indicate that C inputs were largely from roots, either through exudation or death and turnover. The interaction of roots and Fe_p_/Al_p_ also showed that high root inputs, in combination with mineralogy that can adsorb and coprecipitate SOC, may synergize to promote SOC storage and persistence in this disturbed agricultural landscape. These best predictors based on AICc-based model selection also align with developing conceptualizations of soil protection mechanisms^23,41^.

Though the Fe_p_/Al_p_ ratio is exploratory, it is widely understood that sorption and coprecipitation interactions between SOM and Fe oxides, oxyhydroxides, and short-range-ordered (SRO) phases can protect SOC from microbial decomposition^42,43^. It has also been established that short-term redox fluctuations can generate SRO Fe mineral phases that promote SOC decay or storage based on Fe oxidation state^44–46^. Fe(II) is associated with the Fenton reaction, the development of hydroxyl radicals, and increased soil redox potential and SOM decay^47^, while Fe(III) can adsorb and coprecipitate with dissolved organic C^46^. Hansel et al.^48^ further found that the secondary mineralization of ferrihydrite, as the precursor for most iron oxides in soils, is reduced by Al substitution and adsorption. Thus, we suspect that Fe_p_/Al_p_ relates to increased development of stable Fe(III) oxides/oxyhydroxides, as a result of low Al, and that long-term storage of SOC is occurring in this system through the adsorption and coprecipitation of SOC and Fe(III) oxides during secondary mineralization. However, the ratio could also imply that organo-Fe complexes are simply more stable than organo-Al complexes in weathered tropical soils. More detailed investigation into Fe and Al mineralogy in these soils and extracts is needed to resolve a potential mechanism behind Fe_p_/Al_p_.

Models of k_2_ were dominated by Roots which explained most variance while combinations of Fe_c_/Al_c_, Si_p_/Al_p_, and Clay showed weak predictive ability. Though Clay is known to be poorly quantified in tropical soils^49^, Clay has been used as a potential covariate for tropical SOC dynamics^50^ and we found increased Clay, using standard sedimentary procedures, was associated with reduced slow pool decay. In contrast, Fe_c_/Al_c_ and Si_p_/Al_p_ both showed weak association with increased slow pool loss. Increased Fe_c_/Al_c_ may relate to increased Fe redox reactions under anaerobic conditions that can promote lignin decay^47^. However, neither the Fe_c_/Al_c_ or Si_p_/Al_p_ ratios are documented in the literature and further work to resolve if these relationships are meaningful or spurious is needed. Overall, our interpretation of k_2_ is that soils with higher Roots, and associated higher SOC, decay more C based on simple reaction kinetics relating to pool size. Longer incubation experiments to identify and model C with lower turnover is required to resolve the soil parameters associated with the slowest C pools. However, the current data suggests that mechanisms that transport C into slower C pools (e.g., mineral adsorption, coprecipitation, and aggregation) are key, and that once protected in the slower C pools, SOC decays at a much slower and more conserved rate.

Due to the simplicity of the agricultural system and a lack of major SOM or forest litter pools, the confounding influence of free SOC that is not associated with soils was removed. Thus, we expect that the physicochemical soil parameters most associated with SOC, k_2_, and k_1_, fit across depth and contrasting soils, explain the strongest drivers of C response in this tropical agricultural system. The nature of the data and analysis also indicate that the predictors identified by model selection are generalizable through depth and across the landscape, even in the models with weak relationships and low variance explained. Thus, without knowing depth or climate regime we can estimate C dynamics in similar tropical agricultural systems based on several physicochemical soil parameters. Though expanded sampling and ground-truthing at similar locations are needed, these models represent an important conceptual step towards finding generalizable relationships and soil parameters that explain SOC dynamics based on measurable physicochemical properties and representative soil protection mechanisms.

Using the identified soil parameters and models, we plan to test application of ecosystem models in weathered tropical soils throughout the depth profile and eventually assess their predictive ability on SOC stocks. For example, if the identified models and soil parameters are found generalizable and mechanistic, then changes in soil physicochemical parameters should be able to explain differences between SOC improvement studies in tropical agricultural soils. If so, estimates of SOC improvement potential in unstudied tropical agricultural areas could be estimated based on changes in these top physicochemical predictors. This would allow improved estimates of SOC offsets based on improved soil management.

### Exploring depth and rainfall gradients

**Figure 5:**
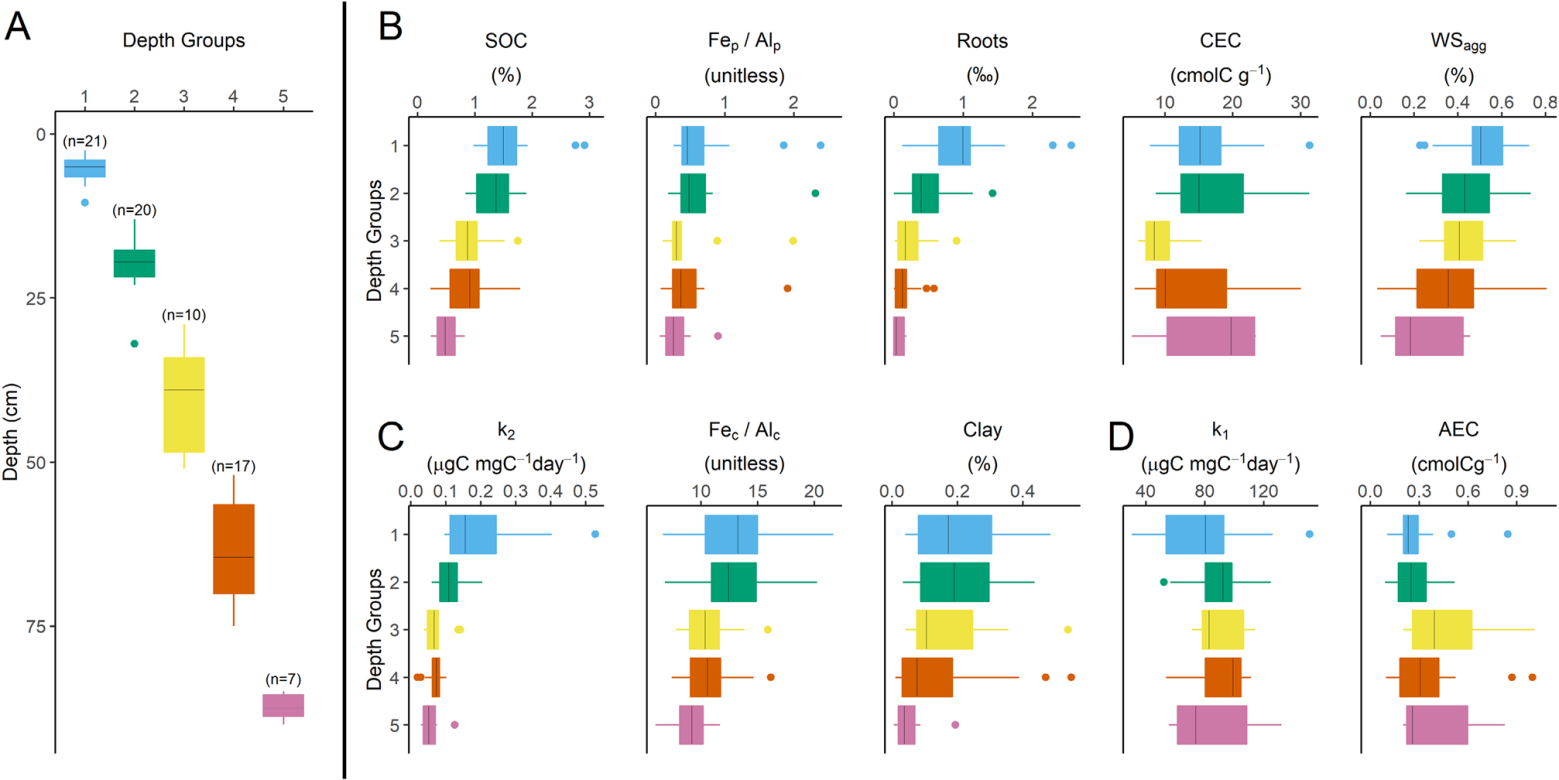
Hierarchical clustering of C response, physicochemical predictors, and depth. Median values, first and third quantiles, and 95% confidence intervals are shown for each hierarchical depth group (**A**), SOC and best physicochemical predictors (**B**), k_2_ alongside best predictors (**C**), and k_1_ and best predictors (**D**).

**Figure 6:**
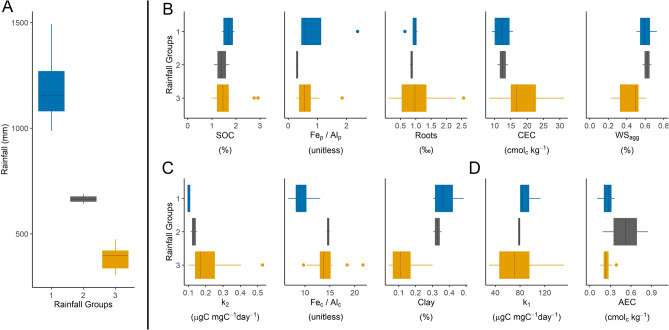
Hierarchical clustering of surface C response, physicochemical predictors, and rainfall. Median values, first and third quantiles, and 95% confidence intervals are shown for each hierarchical depth group (**A**), SOC and best physicochemical predictors (**B**), k_2_ alongside best predictors (**C**), and k_1_ and best predictors (**D**).

Hierarchical clustering was used to connect SOC content and predictive soil parameter values to distinctive groups along gradients of depth (Fig. 5) and rainfall (Fig. 6). Clusters showed contrasting relationships between SOC response, top predictors, and each gradient. As models of SOC content were fit across depth and climate gradients, the top predictors account for differences in C response based on measurable soil parameters established by these gradients over millennial timescales. Though clustering showed clear groups along both depth and rainfall gradients, it is not designed for strong statistical tests. Thus, using hierarchical clustering as a descriptive tool, we discuss relationships between SOC, k_2_, and k_1_ in relation to depth and rainfall gradients as key factors in the development of soil parameters in this system.

Clustering SOC response and predictors by depth to 1 m showed strong reductions in Roots, SOC content, and k_2_ with increasing depth (Fig. 5). Similarly, Clay, WS_agg_, Fe_p_/Al_p_, and Fe_c_/Al_c_ decreased with increasing depth but showed greater variance within each group and weaker association with depth. In contrast, changes in k_1_, CEC, and AEC were not associated with depth. Further when surface soils were clustered by rainfall, several clear rainfall groups were found (Fig. 6). Increased rainfall was not associated with increased Roots or SOC in surface soils. In contrast, k_2_ showed small increases with low rainfall, while k_1_ showed small increases with high rainfall. However, both k_2_ and k_1_ had high variance that limit comparisons in the driest areas. Increased Clay and WS_agg_, and reduced CEC, showed the strongest associations with increased rainfall in surface soils. These patterns align with weathering of clay minerals towards Fe and Al oxyhydroxides.

Hierarchical clustering also demonstrated that rainfall, as an important climate gradient, was not associated with the development of SOC and Roots. Thus, assumption that soil parameters are major controls of SOC storage and dynamics compared to climate were confirmed in this constrained system. Clustering by depth and rainfall also highlights that WS_agg_ and Clay develop with high rainfall and in surface soils that have greater climate interaction compared to deeper soil profiles. Increased rainfall was also associated with decreased CEC in surface soils. In contrast to CEC, changes in Fe_p_/Al_p_ and Fe_c_/Al_c_ were only associated with depth, which suggests that soil forming factors other than rainfall, like parent material and biological interactions are likely involved in the development of these predictive mineral ratios.

## Conclusion

Increased negative surface charge, water stable aggregation, organo-Fe complexation, and root inputs are key SOC protection mechanism across this tropical agricultural system despite high soil disturbance. Root-mineral interactions also synergized to store more SOC. In contrast, no clear mechanisms described slow pool decay rate, with more roots related to more slow pool loss. However, fast pool decay was reduced in areas with negative surface charge. Increased negative surface charge, though a weak effect, may compound with time to shuttle SOC onto soil surfaces and into SOC pools with slower turnover time. Once adsorbed or coprecipitated on mineral surfaces, SOC in slower pools may decay based on simple reaction kinetics, like pool size and temperature. However, incubating soils for more than 90-days is needed to assess the slowest moving SOC pools and any associated soil physicochemical parameters and mechanisms.

Testing soil physicochemical predictors across continuous SOC response, depth, and climate allowed generalization across these gradients, giving SOC estimates created here direct application in similar tropical soils with a history of intensive cane cultivation. In contrast, SOC dynamics predicted by 2-pool incubation models were less effective, though it has not been resolved how weak effects of negative surface charge over long time-periods may drive SOC storage. Our ability to estimate SOC response in post-sugar soils, which represents most agricultural land in the State of Hawaii, will improve with testing the effectiveness of identified models and predictors in other areas, and experiments that test mechanisms and causality. Exploring how identified predictors can extend estimates from soil improvement studies into other areas, and inform underlying relationships in earth system models, could improve our understanding of SOC storage mechanisms and ability to estimate SOC outcomes globally.

## Data Availability

Soil respiration data are available at: https://doi.pangaea.de/10.1594/PANGAEA.943160. Soil carbon dynamics and physicochemical data are available at: https://doi.pangaea.de/10.1594/PANGAEA.943118.
